# A network-based systems genetics framework identifies pathobiology and drug repurposing in Parkinson’s disease

**DOI:** 10.21203/rs.3.rs-4869009/v1

**Published:** 2024-10-14

**Authors:** Lijun Dou, Zhenxin Xu, Jielin Xu, Chang Su, Andrew A. Pieper, Xiongwei Zhu, James B. Leverenz, Fei Wang, Jeffrey Cummings, Feixiong Cheng

**Affiliations:** 1 Cleveland Clinic Genome Center, Lerner Research Institute, Cleveland Clinic, Cleveland, OH 44195, USA; 2 Genomic Medicine Institute, Lerner Research Institute, Cleveland Clinic, Cleveland, OH 44195, USA; 3 Department of Population Health Sciences, Weill Cornell Medical College, Cornell University, New York, NY 10065, USA; 4 Department of Psychiatry, Case Western Reserve University, Cleveland, OH, USA; 5 Brain Health Medicines Center, Harrington Discovery Institute, University Hospitals Cleveland Medical Center, Cleveland, OH 44106, USA; 6 Geriatric Psychiatry, GRECC, Louis Stokes Cleveland VA Medical Center, Cleveland, OH 44106, USA; 7 Institute for Transformative Molecular Medicine, School of Medicine, Case Western Reserve University, Cleveland, OH 44106, USA; 8 Department of Neurosciences, Case Western Reserve University, School of Medicine, Cleveland, OH 44106, USA; 9 Department of Pathology, Case Western Reserve University, School of Medicine, Cleveland, OH 44106, USA; 10 Department of Molecular Medicine, Cleveland Clinic Lerner College of Medicine, Case Western Reserve University, Cleveland, OH 44195, USA; 11 Lou Ruvo Center for Brain Health, Neurological Institute, Cleveland Clinic, Cleveland, OH 44195, USA; 12 Chambers-Grundy Center for Transformative Neuroscience, Department of Brain Health, School of Integrated Health Sciences, UNLV, Las Vegas, Nevada 89154, USA

**Keywords:** Drug repurposing, Electronic Health Record (EHR), Genome-Wide Association Studies (GWAS), Parkinson’s disease (PD), Quantitative trait loci (QTL), Protein-protein interactions (PPI), Risk genes

## Abstract

Parkinson’s disease (PD) is the second most prevalent neurodegenerative disorder. However, current treatments are directed at symptoms and lack ability to slow or prevent disease progression. Large-scale genome-wide association studies (GWAS) have identified numerous genomic loci associated with PD, which may guide the development of disease-modifying treatments. We presented a systems genetics approach to identify potential risk genes and repurposable drugs for PD. First, we leveraged non-coding GWAS loci effects on multiple human brain-specific quantitative trait loci (xQTLs) under the protein-protein interactome (PPI) network. We then prioritized a set of PD likely risk genes (pdRGs) by integrating five types of molecular xQTLs: expression (eQTLs), protein (pQTLs), splicing (sQTLs), methylation (meQTLs), and histone acetylation (haQTLs). We also integrated network proximity-based drug repurposing and patient electronic health record (EHR) data observations to propose potential drug candidates for PD treatments. We identified 175 pdRGs from QTL-regulated GWAS findings, such as *SNCA*, *CTSB*, *LRRK2, DGKQ*, *CD38* and *CD44*. Multi-omics data validation revealed that the identified pdRGs are likely to be druggable targets, differentially expressed in multiple cell types and impact both the parkin ubiquitin-proteasome and alpha-synuclein (a-syn) pathways. Based on the network proximity-based drug repurposing followed by EHR data validation, we identified usage of simvastatin as being significantly associated with reduced incidence of PD (fall outcome: hazard ratio (HR) = 0.91, 95% confidence interval (CI): 0.87–0.94; for dementia outcome: HR = 0.88, 95% CI: 0.86–0.89), after adjusting for 267 covariates. Our network-based systems genetics framework identifies potential risk genes and repurposable drugs for PD and other neurodegenerative diseases if broadly applied.

## Introduction

Parkinson’s disease (PD) is a devastating and complex neurodegenerative disorder that occurs in around 1% of people over the age of 60 [1]. Worldwide, PD has afflicted more than 6.2 million people as of 2015 and is projected to exceed 12 million by 2040[1]. Patients with PD exhibit both motor impairments (e.g., tremors, muscle rigidity and bradykinesia) [2, 3] and non-motor symptoms (e.g., cognitive impairment, sleep disturbances, anxiety, and depression) [4]. Current therapeutics primarily aim to alleviate these symptoms and improve quality of life, such as levodopa to boost dopamine (DA) levels [5]. Despite numerous clinical trials, however, we still lack treatments that slow or stop the disease progression [6]. With our rapidly expanding growing population, there is an urgent need to develop these types of disease-modifying therapies for PD.

Large-scale genome-wide association studies (GWAS) have identified more than 90 independent risk signals for PD across 78 loci, such as *SNCA*, *LRRK2*, *GBA1* (also known as *GBA*) and *MAPT* [7]. However, the majority of reported GWAS variants resided in non-coding regions with unknown function. Molecular quantitative trait loci (xQTLs), such as expression QTLs (eQTLs), protein QTLs (pQTLs), splicing QTLs (sQTLs), methylation QTLs (meQTLs), and histone acetylation QTLs (haQTLs), are crucial functional genomics tools used in the field to identify genes with high probability of being causally linked to a disease. However, we are still lacking effective approaches to interpret the functional relevance of GWAS loci [8, 9]. Recent advances of network methods and deep learning (DL) shows great promise in inferring disease-associated genes and pathways for therapeutic development in Alzheimer’s disease (AD) and other neurodegenerative diseases [10–15]. Therefore, unique integration of the functional effects of non-coding loci via various xQTL elements using network and DL-based models could help discovery of potential disease risk genes in ways that lead to identification of new therapeutic approaches, as demonstrated by recent studies [16–18].

**Figure 1:**
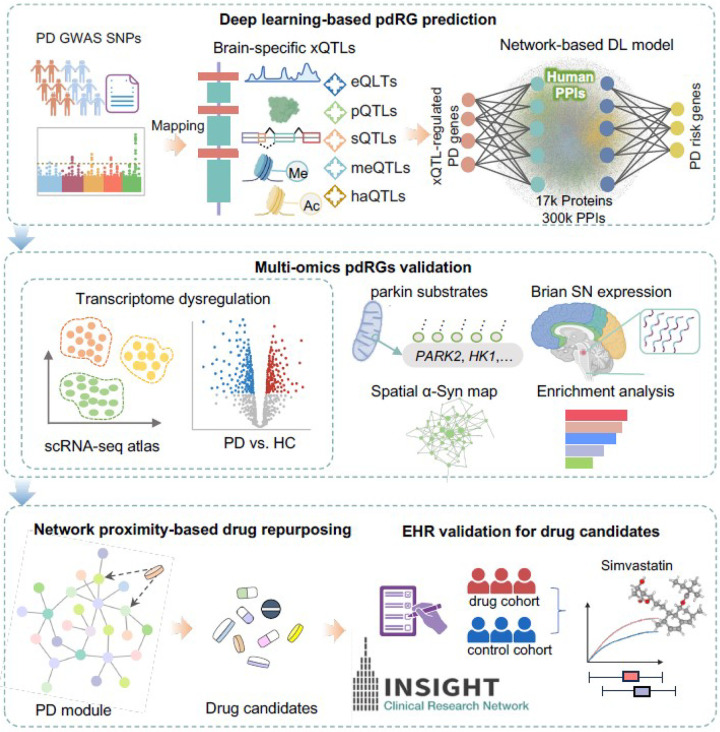
A diagram of genome-wide xQTL-associated risk gene prediction and drug repurposing in PD, created with BioRender.com. It comprises four steps: (1) Deep learning (DL)-based pdRGs prediction by integrating GWAS findings and five types of xQTL findings, including expression QTLs (eQTLs), protein QTLs (pQTLs), splicing QTLs (sQTLs), methylation QTLs (meQTLs), and histone acetylation QTLs (haQTLs); (2) multi-omics validation of putative pdRGs through single nuclei transcriptome data, parkin-dependent substrates, alpha-synuclein (*α*-syn) modifiers, etc; (3) network proximity-based drug repurposing within human protein-protein interactome; and (4) drug validation via real-world EHR data.

In this study, we present a network-based systems genetics framework to identify potential disease-associated genes for PD from large-scale genetics and xQTL findings (Figure 1, *cf.* Methods). Specifically, we leverage non-coding PD loci effects from GWAS across five types of brain-specific xQTLs. The fundamental premise of our systems genetics framework is that PD likely risk genes will exhibit distinct functional characteristics compared to non-risk genes, thereby rendering them able to be distinguished by their aggregated xQTL features under the human protein-protein interaction (PPI) network. With this approach, we identified a set of PD-associated genes that are highly expressed (enriched) in well-known PD pathways and likely to represent known drug targets. We then combined network proximity-based drug prediction and large-scale real-world patient data (RWD) validation to identify repurposable drugs (i.e., simvastatin) for potential prevention or treatment of PD.

## Methods

### Collection and Mapping of PD GWAS and brain-specific xQTL data

**Figure 2:**
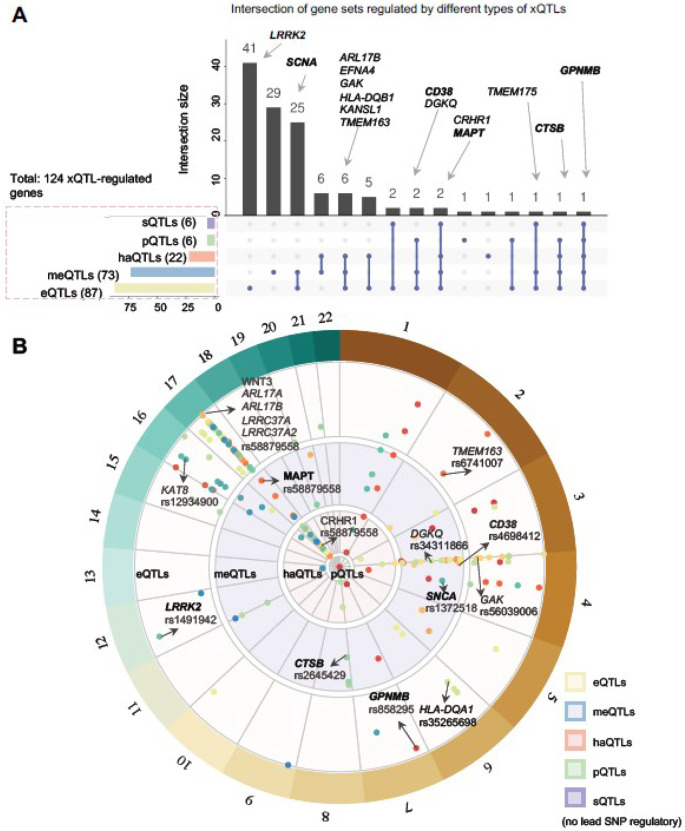
Landscape of PD GWAS loci regulated by five types of molecular xQTL data. (A) Upset plot illustrating the intersection of gene sets regulated by different types of xQTLs. The bar plot on the left depicts the size of gene sets linked to each xQTL data, while the bar plot on the top shows the intersection size of gene sets regulated by multiple types of xQTLs. Well-known PD genes are labeled in bold. (B) Landscape of GWAS loci (GRCh37, LD clumping r^2^_cutoff=0.1) for five types of xQTL-regulated genes, including eQTLs, meQTLs, haQTLs, pQTLs and sQTLs (i.e., 178 connection between 85 genes and 50 lead SNPs) from outside to inside (Methods and Materials, **Tables S1**).

To obtain more sufficient PD genetic data, we adopted a mapping strategy to obtain GWAS loci linked genes by incorporating multiple types of brain-specific regulatory elements. First, we collected 5,818 suggestively significant SNPs (*p* < 1×10^−5^) from two recent PD-related GWAS studies (one is related PD risk, study ID: GCST009325) [7] and one is related PD age at onset (AAO), study ID: GCST007780 [19]). Then, we considered five types of brain-specific molecular regulatory elements. Specifically, eQTL data is extracted from Sieberts et al. [20] (including cortex and cerebellum regions) and Klein et al.[21] (considering basal ganglia, cerebellum, cortex, hippocampus and spinal cord regions in this study). And pQTL data is from Robins et al. [22] and Wingo et al. [23], and sQTLs from Qi et al. [24]. Other two types of QTLs (meQTLs and haQTLs) are both collected from Ng et al. [25]. During the mapping procedure for eQTLs, sQTLs and pQTLs, we retained only regulated genes for which linked SNPs appeared simultaneously in GWAS and QTL sources. As for meQTLs and haQTLs, we separately mapped the genome-wide peaks and methylation regions to the nearest gene (closest function in bedtools [26]). Here, we screened only protein-coding genes via ensemble gene reference annotation GRCh37.87 for downstream analysis. In total, we obtained 42,859 genome wide regulatory connections, (Figures 2 and Supplementary **S1**), corresponding to 124 regulated genes (Figures 2, Supplementary **S1** and **Table S1**). When considering LD correction, only 128 independent SNPs can be filtered by LD clumping (plink v1.9, 1000 Genomes (European ancestry), [LD r^2^_cutoff = 0.1, window size = 1MB]) from these two GWAS studies, contributing to 178 regulatory connections (i.e., 85 regulated genes, Figure 2B). Circular plot of PD GWAS loci regulated by these xQTLs was created by BioCircos [27] and locus zoom plot for *GPNMB* locus by locuszoomr [28].

### Collections of known PD-associated genes for model validation

To evaluate model performance, we collected PD-associated genes from the Target Illumination GWAS Analytics (TIGA) database [29]. This prioritized multiple targets by leveraging gene-trait association with multiple studies and evidence. Here we filtered 127 PD risk genes with meanRankscores > 70 (Supplementary **Table S2**).

### Building human protein–protein interactome

Assembling 15 widely used PPI datasets, we built a comprehensive human protein–protein interactome [15, 16], including (1) binary PPIs validated by high-throughput yeast-two-hybrid (Y2H) system [30]; (2) kinase-substrate interactions; (3) signaling networks by SignaLink2.0 [31]; (4) binary PPIs based on the three-dimensional (3D) structures from Instruct [32]; (5) protein complexes data (~56,000 candidate interactions) from BioPlex V2.01 [33]; and (6) carefully literature-curated PPIs which are identified by affinity purification followed by mass spectrometry (AP-MS) or literature-derived low-throughput experiments. In total, the established network consists of 351,444 PPIs among 17,706 distinct proteins (https://alzgps.lerner.ccf.org). The largest connected component (LCC) was extract using the igraph package (https://igraph.org/), including 336,549 PPIs covering 17,456 proteins.

### Network-topology-based PD likely risk gene prediction

To better translate the human genetic findings to pathobiology and therapeutic discovery, Xu et al. proposed a network topology-based DL framework (NETTAG) and applied this in AD [16]. The preliminary premise of NETTAG is that comparison of risk to no-risk genes should reveal distinct functional attributes and thereby distinguish by aggregating genomic features under the human protein interactome, like xQTLs, enhancers, and CpG islands. In this study, we extended NETTAG[16] by incorporating 2^nd^ order neighbors for node representation. This is mainly due to 2 reasons: 1) Previously, we found that nodes and their 2^nd^ order neighbors most likely shared the same gene ontology (GO) annotations [16]; 2) The PPI contains multiple triangles. This observation indicates that designing graph neural network methods that incorporate higher-order neighbors may not be effective, as higher-order neighbors are often the same as 1^st^ and 2^nd^ order neighbors. This redundancy reduces the value of considering higher-order connections. Thus, we have now extended NETTAG [16] by incorporating 2^nd^ order neighbors for node representation as followings,

(1) $$H^{\left(i+1\right)}=ReLU\left(\sum_{j=0}^{2}\;{\overset{ˆ}{A}}^{j}H^{\left(i\right)}W_{j}^{\left(i\right)}\right)$$

Here, $H^{\left(i\right)}$ represents the node presentation at layer i. $\overset{ˆ}{A}$ is the symmetrical normalization of the adjacency matrix A, i.e., $\overset{ˆ}{A}=D^{-1/2}AD^{-1/2}$, D is the degree matrix. Additionally, we incorporated the jumping knowledge (JK) network [34], which was developed to adaptively utilize varying extents of the surrounding network for each node, thereby enhancing the capacity for representations that more fully consider the underlying structure as followings,

(2) $${\overset{‾}{H}}^{\left(L\right)}=\left[H^{\left(1\right)}\|\cdots \|H^{\left(L-1\right)}\|H^{\left(L\right)}\right]$$

Utilizing the mapped xQTL-regulated genes as input, we trained this model based on the well-established human PPI network. In order to improve the stability of prioritized proteins, we ensembled the prediction results from 10 random seeds. Subsequently, we validated them using TIGA findings[29].

### Transcriptome dysregulation of pdRGs at single-nuclei resolution

To verify transcriptome dysregulation of pdRGs, we utilized one snRNA-seq dataset GSE178265 within the human SN region [35], GSE178265, which covers 387,558 nuclei from ten PD/LBD cases and eight healthy controls (HC). First, we removed low-quality nuclei [nFeature_RNA: 200~10,000, nCount_RNA: 650~ 30000, percent.mt < 10, percent.hb < *5*, percent.ribo *<* 5]. Then, we implemented DoubletFinder [36] to predict potential heterotypic doublets or triplets. Afterwards, we retained 288,988 high-quality nuclei to explore transcriptome alterations, in which the batch effect from different samples was corrected by implementing Harmony [37]. With annotation by canonical marker genes, these nuclei were grouped into seven main clusters, including DA neurons with markers of *SLC18A2, SLC6A3* and *TH*), non-DA neurons (*RBFOX3*), astrocytes (Astro: *AQP4* and *GJA1*), microglia (MG: *C3*, *P2RY12* and *CX3CR1*), oligodendrocyte precursor cells (OPC: *VCAN* and *OLIGI*), oligodendrocytes (ODC: *MOBP* and *MOG*), endothelial cells/pericytes/fibroblasts (Endo_Peri_Fib: *CLDN5*, *FLT1*, *COL1A2* and *MGP*) (Figures 4 and S1D). Cell type specific DEGs (PD vs. HC) were calculated using FindMarkers [Wilcoxon rank sum test: min.pct= 0.01, |log2FC| > 0.25 and *p* < 0.05]. Furthermore, we explored gene expression patterns by comparing DA neurons and non-DA nuclei in PD patients’ brain (Figures 4, S1 and Supplementary **Table S5**). The snRNA-seq analysis was performed in Seurat v3 [38].

### PD/LBD-associated genes from GWAS studies

GWAS studies have accumulated various mutations associated with PD and LBD. The association file (v1.0) was downloaded from GWAS catalog (https://www.ebi.ac.uk/gwas/). Subsequently, we filtered 157 reported genes related PD genes using two keywords “Parkinson” and “Lewy body” with *p* < 5×10^−8^.

### Gene expression profiles of substantia nigra

In this stage, we evaluated the gene expression specificity of pdRGs in 33 human brain tissues using the RNA-seq data from GTEx database (v8) [39]. Based on transcript per million counts (TPM), the expression specificity of gene i in tissue t is defined as

(3) $$z_{it}=\frac{E_{it}-E_{i}}{\sigma_{i}}$$

where $E_{i}$ and $\sigma_{i}$ are the mean and standard deviation of the expression of gene i across all considered tissues, respectively. $E_{it}$ indiates the mean expression of gene i in tissue t. We retrieved highly expressed genes in SN with Z>0 (Z=0 if Z<0, Supplementary **Figure S1**).

### Construction of pdRGs-induced PPI network for PD

To characterize the interaction patterns among pdRGs, we mapped them with LCC, including a PD disease module with 258 edges among 119 nodes. After removing self-loops and isolated edges, the PPI module for PD covered 191 edges (PPIs) among 103 nodes (pdRGs) (Figure 5). The network was visualized by Cytoscape software [40].

### Network-proximity-based Drug discovery

Network proximity [41] defined a closet distance d(s,t) between disease protein s(s∈S) and drug target t(t∈T) by calculating the average length of shortest path in the human protein-protein interactome as followings,

(4) $$d(S,T)=\frac{1}{∥T∥}\sum_{t\in T}\;{min}_{s\in S}\;d(s,t)$$

Subsequently, Z score was applied to quantify the significance of network proximity (i.e., distance) as followings,

(5) $$Z=\frac{d-\mu }{\sigma }$$

Here, μ and σ are the mean and standard deviations of permutation tests (1,000 random experiments), which help reduce literature bias associated with well-studied proteins. As a result, we applied Z<-3,p<0.05 and screened only FDA-approved drugs, and then retained 70 drugs for PD treatment from 2,938 candidates.

### Real world EHR validation of repurposed PD drugs

To validate drug candidates, we implemented real-world data (RWD) from INSIGHT, containing 15 million patients in the New York City metropolitan area and Hoston (Figure 7). Use of INSIGHT data was approved by the Institutional Review Board (IRB) of Weill Cornell Medicine under protocol 21–07023759. This included 5,703 fall-diagnosed PD patients and 13,244 dementia-diagnosed PD patients from January 2000 to September 2022.

#### Eligibility criteria.

Patient eligibility criteria for analysis included (Figure 7):

1. Patients should have at least one PD diagnosis according to International Classification of Diseases 9th and 10th revision codes (ICD-9/10, Supplementary **Table S8**), including 332.0 (ICD-9) and G20 (ICD-10).
2. Patient’s age was >= 50 years old at the first PD diagnosis.
3. Patients who had neurodegenerative disease diagnoses before his/her first PD diagnosis was excluded.

#### PD outcomes.

We considered PD related outcomes including dementia and falls (indicating advanced motor impairment and dyskinesia), which were defined by ICD-9/10 diagnosis codes. Drug treatment efficiency was defined by reducing the risk to develop the PD outcomes.

#### Follow-up.

Each patient was followed from his/her baseline until the day of the first PD outcome event, or loss to follow-up (censoring), whichever happened first (Supplementary **Table S9**).

#### Trial Emulation.

We first obtained DrugBank ID of each tested drug and translated it to RxNorm and NDC codes using the RxNav API (application programming interface). Drugs which were used by less than 100 patients were excluded for analysis. Following Ozery-Flato et al. [42], we defined the PD initiation date of each patient as six months prior to his/her first recorded PD diagnosis event. This accounted for the likelihood that PD may be latently present before formal diagnosis. We defined the index date as the beginning time of treatment of a tested drug candidate or its alternative treatment. We also defined the baseline period as the time interval between the PD initiation date and the index date for each patient. We required that: (1) The index date was later than the PD initiation date. (2) Onset of PD outcomes were later than the index date. Then, for each tested drug, we built an emulated trial using the following procedures:

1. We built its treated group as the eligible PD patients who took the tested drug after PD initiation;
2. We built a control group as:
  - Patients who received alternative treatment of the tested drug, i.e., drugs from a same Anatomical Therapeutic Chemical level 3 [ATC-L3] classification of the tested drug, excluding the drug itself.
  - To control confounding factors, we performed the propensity score matching as described below.

#### Propensity score matching (PSM).

We collected three types of covariates at the baseline period for each patient: 1) We included 64 comorbidities including comorbidities from Chronic Conditions Data Warehouse and other risk factors that were selected by experts [43]. The comorbidities were defined by a set of ICD-9/10 codes. 2) We considered usage of 200 most prevalent prescribed drug ingredients as covariates in this analysis. These drugs were coded using RXNORM and grouped into major active ingredients using Unified Medical Language System. 3) We also included other covariates, including age, gender, race, and the time from the PD initiation date to the drug index date. In total, we included 267 covariates for analysis, including 2 continuous variables (age and PDToDrug, i.e. the time from the PD diagnosis date to the initiation date of the targeted drug) and 265 binary variables (comorbidities, medications, and gender). There are no missing values for age, PDToDrug, or gender. For the binary variables related to comorbidities (64 variables from ICD-9/10 codes) and medications (200 variables from RXNORM codes), we applied 0 to fill in missing values, indicating that the patient did not have the comorbidity or take the medication.

For each emulated trial, we used a propensity score framework to learn the empirical treatment assignment given the baseline covariates and used an inverse probability of treatment weighting to balance the treated and control groups [44]. For each trial, a 1:1 nearest-neighbor matching was performed to build the matched control group [44]. The covariate balance after propensity score matching was assessed using the absolute standardized mean difference (SMD) [45]. For each covariate, it was considered balanced if its SMD≤0.2, and the treated and control group were balanced if only no more than 2% covariates were not balanced [46]. To enhance robustness of the analysis, we created 100 emulated trials for each tested drug. Tested drugs that had <10 successfully balanced trials were excluded for analysis.

#### Treatment effect estimation.

For each tested drug, we estimated drug treatment effect for each balanced trial by calculating the hazard ratio (HR) using a Cox proportional hazard model [47], comparing the risk to develop a specific outcome between the treated and control groups. We reported the median HR with 95% confidence intervals (CI) obtained by bootstrapping [48]. A hazard ratio < 1 indicated the tested drug can reduce risk to develop a specific outcome and a P value < 0.05 was considered as statistically significant. The trial emulation pipeline for treatment effect estimation was implemented using Python packages psmpy[49] for propensity score framework and lifelines[50] for the Cox proportional hazard model.

### Statistical analysis

During the multi-omics validation of pdRGs, we applied Fisher’s test to examine whether the proposed pdRGs were significantly enriched in the relevant evidence gene set. We took all genes (~17k) involved in human interactome network as background for this statistic test (significant if *p* < 0.05). This was executed on an R program.

## Results

### A network-based systems genetics framework to infer PD-risk gene

As illustrated in Figure 1, we developed a network-based deep learning (DL) framework to infer putative PD risk genes (pdRGs) and predict repurposable drugs for PD from genetics and human brain-specific functional genomic findings. This framework assembles PD non-coding GWAS loci effects across five types of human brain-specific xQTL profiles and the human PPIs using a deep learning approach (*cf.* Methods). The procedure was divided into four steps. First, we utilized a DL model to cluster PPIs into multiple functional network modules by capturing its topological structures within the human PPIs. Second, we quantified a node’s (gene’s) score by integrating its functional similarity with each identified gene with brain-specific xQTL evidence via influencing non-coding loci from PD GWAS loci. Third, we prioritized pdRGs by aggregated gene regulatory features from the xQTL evidence [16] and validated those pdRGs using multi-omics approaches, including transcriptome dysregulation from single-nuclei RNA sequencing (snRNA-seq) data from human brains with PD, parkin-dependent substrates, alpha-synuclein (*α*-syn) modifiers, gene expression in human brain substantia nigra (SN) region, and pathway enrichment analysis (*cf.* Methods). Fourth, we applied network proximity-based method to predict candidate repurposable drugs for PD, which were further validated using large-scale electronic health record (EHR) patient data (*cf.* Methods).

### PD GWAS loci are enriched in human brain xQTLs

**Figure 3:**
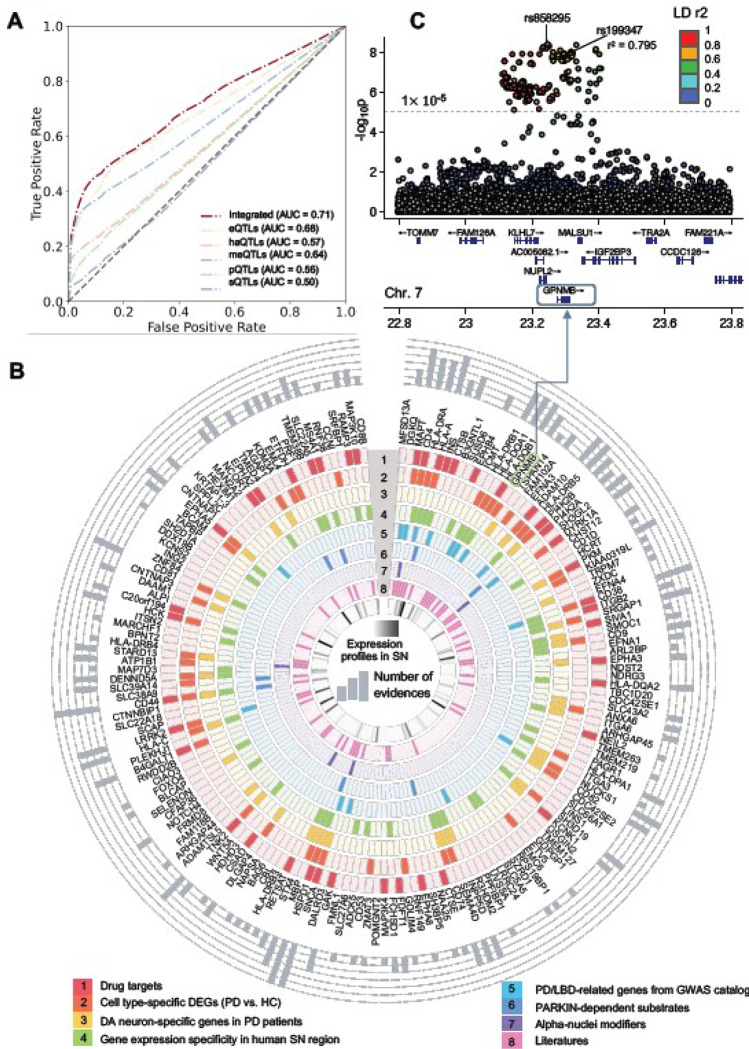
Prediction of pdRGs based on the network-topology DL framework and validation using multi-omics data. (A) Model performance (i.e., AUCROC) for PD risk gene prediction. The dark red line represents the predicted score for our final model utilizing aggregated xQTL data. The other colored lines depict the respective model performance with single xQTL data. (B) Circular plot showing eight types of evidence for identified 175 pdRGs, which are displayed clockwise from high to low according to the predicted Z score. Validations are illustrated from outside to inside, including (1) Drug targets; (2) Cell type-specific differentially expressed genes (DEGs, PD vs. HC); (3) DA neuron-specific genes when comparing DA neurons and non-DA cells in PD patients’ brains (4) Gene expression specificity in human substantia nigra (SN) region (Z>0); (5) PD/LBD-associated genes from GWAS catalog; (6) PARKIN-dependent substrate; (7) alpha-synuclein map; and (8) literatures. Expression profiles of pdRGs in SN are shown in the innermost circle colored by Z score from GTEX database (Z = 0 if Z < 0). The accumulated counts of evidence are plotted in the outermost circle. (C) Locus zoom plot of the predicted pdRGs *GPNMB* [97] colored by LD r^2^ (EnsDb.Hsapiens.v75, window_size=1Mb).

We assembled two recent large PD GWAS studies [7, 19] and then mapped GWAS variants (*p* < 1×10^−5^) with five types of human brain-specific gene regulatory elements, including eQTLs [20, 21], pQTLs [22, 23], sQTLs [24], meQTLs and haQTLs [25] (*cf.* Methods). As a result, we pinpointed 124 protein-coding genes that are regulated by at least one type of genome-wide xQTLs (Figures 2, **Supplementary Table S1** and **Figure S1**). As depicted in Supplementary **Figure S1A**, eQTLs contribute to 27,165 connections between genes and GWAS SNPs (63.38%), followed by haQTLs (10,924, 25.49%), meQTLs (4,697, 10.96%), pQTLs (52, 0.12%), sQTLs (21, 0.05%). After considering linkage disequilibrium (LD, *r*^2^ > 0.1, window =1Mb) correction, we found that 85 of 124 (~ 68.5%) mapped genes are linked to lead SNPs. Proxy SNPs (r^2^ > 0.8) also contributed another 37 linked genes for downstream analysis (Supplementary **Figure S1B**). Figure 2A shows the intersection of gene sets regulated by different xQTLs. Specifically, *GPNMB* is regulated by all five types of xQTLs (i.e., eQTLs, haQTLs and meQTLs via lead SNP rs858295 [*p* = 3.83×10^−9^], and pQTLs and sQTLs via multiple proxy SNPs, such as rs199347 [*p* = 1.52×10^−8^, r^2^ = 0.795])[7] (Figure 3C). Notably, the eQTL effect of rs199347 on *GPNMB* has been previously reported [51], which also exhibited 94% posterior probability of the PD risk. Moreover, three genes were characterized by four types of xQTL evidence, including *CTSB* (eQTLs, meQTLs, haQTLs and pQTLs), *CRHR1* and *MAPT* (eQTLs, meQTLs, haQTLs and sQTLs). For the well-known PD gene *SNCA*, we observed two regulatory elements of eQTLs and meQTLs. For *LRRK2*, only the eQTL effect was observed. In total, 87, 73, 22, 6 and 6 unique genes were found to be regulated by PD GWAS loci through eQTLs, meQTLs, meQTLs, sQTLs, and pQTLs, respectively. Figure 2B illustrates the distribution of mapped PD GWAS loci (178 connections for 85 mapped genes by 50 lead SNPs). We found that SNP variants on chromosome 17 were linked to 18 genes (including *MAPT*, *ARL17A*, *ARL17B*, *CRHR1*, *WNT3*, and *LRRC37A2*, etc.). Particularly, many patients with familial frontotemporal dementia with parkinsonism (FTDP) presented mutations in *MAPT* (microtubule-associated protein tau) [52]. We also found enrichment for chromosome 4 related to 12 genes, such as *SNCA* (rs1372518), *CD38* (rs4698412). Of note, rs1372518 is a significant variant for Lewy body dementia (LBD) from functional mapping and annotation of genetic associations (FUMA) analysis[53]. We additionally identified several other key PD risk genes harboring genome-wide significant loci, enriched by human brain xQTLs as well, including *LRRK2* (rs1491942), *GPNMB* (rs858295), *TMEM163* (rs34311866), and *TMEM163* (rs6741007). Next, we turned to leverage these human brain-specific xQTL data to infer potential PD risk genes under the human protein-protein interactome model.

### Network-based prediction of PD likely risk genes

We implemented a network topology-based deep learning (DL) framework within the human protein interactome to test the effects of PD loci and predict disease-associated genes (*cf.* Methods). First, we employed a network-based self-supervised learning method to classify PPIs into functional subnetwork modules, in which the involved genes share more biological relationships. Next, we inferred likely risk genes by integrating PPI-derived network modules with multimodal analyses of five types of xQTLs implicated by PD GWAS loci. Model performance was further evaluated by comparing results with the PD-related genes from the Target Illumination GWAS Analytics (TIGA) database[29] (Figure 3A). The best area under receiver operating characteristic curve (AUROC) was found to be 0.68 for eQTLs and the AUROC was increased to 0.71 after integrating all evidence from five types xQTL data.

We therefore utilized the integrated model after combining the five types of human brain-specific xQTLs and then identified 175 genes as putative pdRGs (**Table S3** and Figure 3B). This encompassed multiple well-known PD risk genes (such as *SNCA, GBA, GPNMB, LRRK2, CTSB,* and *MAPT*) as well as other candidate genes (such as *MFSD13A*, *DGKQ*, *CD4*, and *HSPD1*). These pdRGs are significantly enriched in known PD-associated gene sets (DisGeNET [*q* = 4.8 ×10^−4^]) (**Figure S1C**). In addition, pdRGs were enriched in other neurodegenerative diseases (e.g., LBD and Dementia in PD [*q* < 0.05]) as well.

### pdRGs are enriched in PD-related pathobiological pathways

To examine PD pathobiological pathways for pdRGs, we assembled eight types of evidence (Figure 3B): (1) known drug targets, (2) cell type-specific differentially expressed genes (DEGs) between PD and healthy controls (HC) (i.e., PD vs. HC) from human brain snRNA-seq data, (3) DA neuron-specific genes between DA neurons and non-DA brain cells in PD patients’ brains; (4) gene expression specificity in SN compared to other human brain regions from the GTEx [39]; (5) genetics-reported PD/LBD-associated genes from the GWAS catalog database [54]; (6) parkin-dependent substrates [55], (7) *α*-syn modifiers [56], and (8) PD-associated genes from the published literatures. Together, 113 of 175 (~64.6%) pdRGs exhibited at least one type of association with PD (bar plot in the outermost circle), such as *SNCA*, *HSPD1* and *CD44* with six types of evidence and *MAPT*, *HLA*-*A* and *CD38* with five types of evidence.

PD is characterized by the progressive loss of DA within the SN, a neurotransmitter involved in reward and movement regulation [57]. We found that pdRGs are more likely to be expressed in SN compared to other brain regions (Fisher’s test, where all genes involved in human protein interactome are treated as background [58] [*p* = 0.016], Supplementary **Figure S1D**) from the GTEx database [39]. For instance, these SN-specific pdRGs are significantly enriched in interferon gamma signaling [*q* = 5.75 ×10^−11^], adaptive immune system [*q* = 5.75 ×10^−11^], and cytokine signaling in immune system [*q* = 3.41 ×10^−8^].

Mutations in *PARK2* (encoded the parkin protein) are the most common risk factors for autosomal recessive PD [59]. It controls mitophagy and programmed cell death via ubiquitination of a variety of proteins (called substrates) [60]. Interestingly, we found that pdRGs are enriched in parkin-dependent substrates [55], including *CD44*, *HLA-A*, *HSPD1*, *SLC22A5*, *SLC39A14*, *EML4*, *PI4K2A*, and *NAA25* (**Table S5**). Modifier of *α*-syn are also known to play essential roles in PD and LBD [61], and we found pdRGs are enriched in the *α*-syn modifiers as well (Fisher’s test: [*p* = 0.047]), including *ATP1B1*, *CBL*, *MAPT*, *SH3GL2*, and *SNCA* [56]. In summary, predicted pdRGs are associated with various PD-related pathobiological pathways, including immune pathways, *α*-syn modifiers, and parkin-dependent substrates [55].

### Cell type-specific transcriptome dysregulation of pdRGs in PD

**Figure 4:**
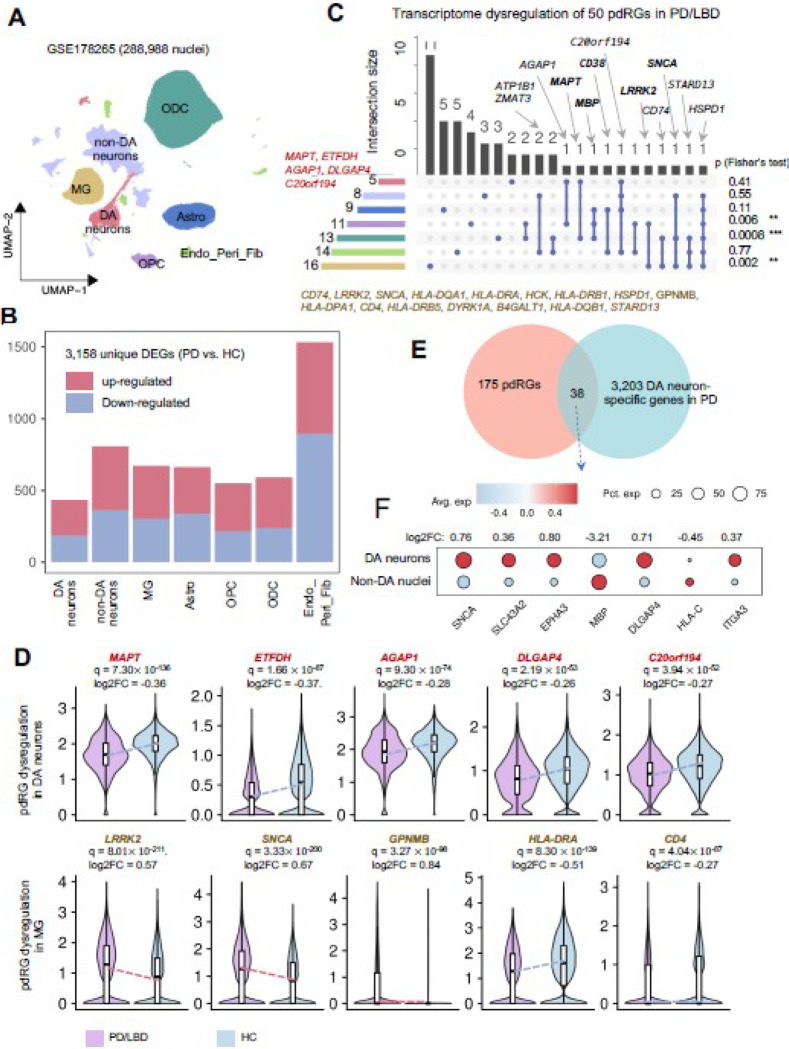
Single-nuclei transcriptome validation of pdRGs within human SN region. (A) Uniform manifold approximation and projection (UMAP) embeddings of seven main cell types of 288,988 nuclei from GSE178265, including dopamine neurons (DA neurons), non-DA neurons, astrocytes (Astro), microglia (MG), oligodendrocyte precursor cells (OPC), oligodendrocytes (ODC), endothelial cells/pericytes/fibroblasts (Endo_Peri_Fib). (B) Stacked bar plot showing the number of cell-type-specific DEGs (PD vs. HC). (C) Upset plot depicting the transcriptome dysregulation of 50 identified pdRGs, along with fisher’s test results on the left (* p <0.05, ** p<0.01, *** p<0.001). The bar plot on the left showed the size of dysregulated pdRGs in different cell types, while the bar plot on the top shows the intersection size of dysregulated pdRG sets observed in multiple cell types. Well-known PD genes are labeled in bold. (D) Transcriptional expression patterns of selected pdRGs in DA neurons (upper panel) and MG (lower panel). (E) Venn plot showing the intersection of pdRGs and DA neuron-specific genes in PD patents. (F) Dot plot of selected seven pdRGs with different expression patterns in DA neurons compared to non-DA nuclei.

We next investigated cell type-specific transcriptome dysregulation of pdRGs in human brains with PD using a large snRNA-seq dataset (*cf.* Methods) (Figure 4 and **Supplementary Figure S1**). Removal of low-quality and potential doublets yielded 288,988 sequenced nuclei across seven major cell types, including DA neurons, non-DA neurons, astrocytes (Astro), microglia (MG), oligodendrocyte precursor cells (OPC), oligodendrocytes (ODC), and endothelial cells/pericytes/fibroblasts (Endo_Peri_Fib). We then identified 3,158 DEGs in PD compared to HC across seven cell types (Wilcoxon Rank Sum test: [|log2FC| > 0.25, *q*< 0.05], Figure 4B and **Supplementary Table S6**). We found that pdRGs are more likely to be differentially expressed in PD brain cells (50 DEGs, Fisher’s test: [*p* = 2.66×10^−6^]).

As illustrated in Figures 4C and 4D, five pdRGs were significantly downregulated in DA neurons, including *MAPT*, *ETFDH*, *AGAP1*, *DLGAP4*, and *C20orf194*. Regarding MG, we observed 16 differentially expressed pdRGs (i.e., *LRRK2*, *SNCA*, *GPNMB*, *HLA-DRA* and *HLA-DRB1*). Beyond DA neurons, there is growing recognition of the microglial function in PD pathogenesis [62], such as *α*-syn-activated microglia [63], microglia and neuron interaction [64]. Specifically, *GPNMB* was upregulated in MG [*q* = 3.27 × 10^−98^, log_2_FC = 0.84] (Figure 4D). A GWAS locus of rs199347 was associated with *GPNMB* through colocalization analyses of eQTL and PD risk loci, confirmed by allele-specific expression (ASE) studies in human brains [51]. *GPNMB* encodes the transmembrane protein glycoprotein nonmetastatic melanoma protein B and co-immunoprecipitated with *α*-syn in cells to influence disease progression [51]. The association of *GPNMB* and increased risk of PD has also been observed in transcriptome-wide association study (TWAS) [65] and Mendelian randomization (MR) analysis [66]. *LRRK2* also showed similar upregulation trends in MG [*q* = 8.01 × 10^−211^, log_2_FC= 0.57]. Several pdRGs (such as *MAPT*, *MBP*, *CD38*, *LRRK2*, *SNCA*) are differentially expressed in more than one cell type. For example, *HSPD1*, encoding heat shock protein family D (Hsp60) member 1, is differentially expressed across all six major brain cell types except DA neurons. *HSPD1* has been reported as a critical chaperone via regulating proteostasis of *α*-syn (preventing α-synuclein aggregation) and parkin (involved in mitochondrial protein folding) [67]. In summary, Fisher’s test supported the observation that the identified pdRGs are significantly enriched in DEGs across MG [*p* = 0.002], ODC [*p* =0.0008] and OPC [*p* = 0.006] [67] from the PD patients’ brains, revealing potential functional roles in PD pathobiology by a cell type-specific manner.

We further compared the gene expression patterns in DA neurons to other brain cell types in PD brains (Figures 4E and 4F). We found 38 pdRGs that presented distinct expression [p=0.02], including *SNCA*, *SLC43A2*, *MBP*, etc. For example, *SNCA* is more likely to be expressed in DA neurons than non-DA cells [*p* = 0, log2FC = 0.76]. In total, 64 of 175 pdRGs showed transcriptome changes across multiple cell types in the SN of PD patients.

### PD network module identified from the protein-protein interactome

**Figure 5:**
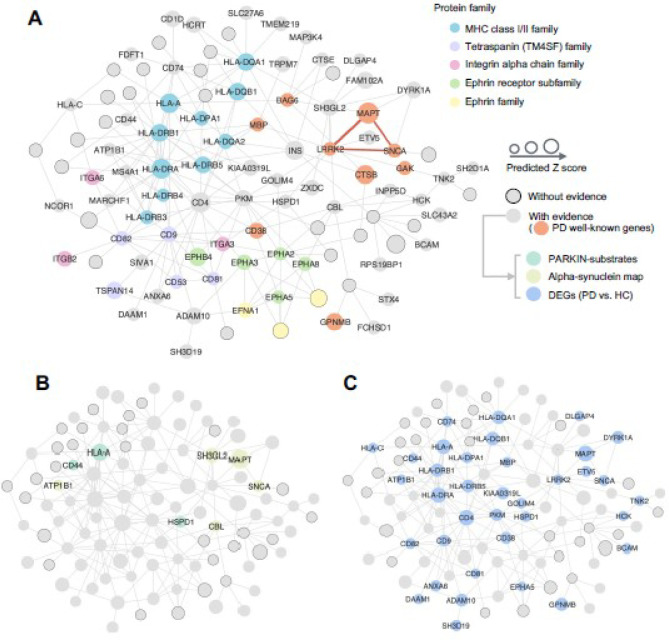
Visualization of pdRGs-derived protein-protein interactome (PPI) network for PD. (A) The overall PD module covering 191 non-isolated PPI pairs (edges) within 103 pdRG-encoded proteins (nodes). All pdRGs with existing evidence are labeled by protein symbol without border (where orange nodes gave well-known PD risk genes), while the size illustrates the predicted score. Parts of nodes were colored by the protein family they belong to. Three well-known pdRGs formed a clique (*LRRK2*-*SNCA*-*MAPT*, red edges in bold). (B-C) Source of three types of external evidence for pdRGs, including parkin substrates and α-syn network (B) and cell-type specific DEGs (PD vs. HC).

To better understand PD pathogenesis, we further investigated network topology for the proteins encoded by the identified pdRGs within the human protein-protein interactome. After removing self-loops (homodimers) and isolated nodes, the pdRGs formed a disease module comprising 191 edges (PPIs) among 103 nodes (pdRGs) (Figure 5A) containing several known key genes for PD, such as *SNCA*, *MAPT*, *LRRK2*, and *CD38* [68]. In total, 75 pdRGs involved in this network module were found to be associated with PD (**Supplementary Table S6**).

Figure 5B highlighted pdRGs enrichening in parkin-dependent substrates (*CD44*, *HLA*-*A* and *HSPD1*) and *α*-syn modifiers (pdRGs, *ATP1B1*, *CBL*, *SH3GL2*, *MAPT* and *SNCA*) in the PD network module. Meanwhile, Figure 5C highlighted 36 pdRGs with significantly changed expression patterns across multiple brain cell types from snRNA-seq data analysis (PD vs. HC). We noted that pyruvate kinase (*PKM*) played a crucial role in this disease network by interacting with 14 pdRGs. In non-DA neurons, *PKM* is significantly upregulated in PD patients compared with HC (log_2_FC = 0.80, *q* =0). It has also recently been shown that *PKM* was destabilized by loss of *UCHL1* (also known as *PARK5*), which mitigates PD-related phenotypes in drosophila and mammalian cells [69]. Similarly, *CD4* (downregulated in MG [q = 4.04× 10^−87^, log2FC = −0.27]) also kept high connectivity with other pdRGs and was downregulated in MG [log2FC = 0.27]. Notably, a PD mouse model found that *CD4* mediated neuroinflammation and neurodegeneration [70]. Taken together, pdRGs are significantly enriched in various PD-related pathological pathways.

### Network proximity-based discovery of candidate drugs for PD.

**Figure 6:**
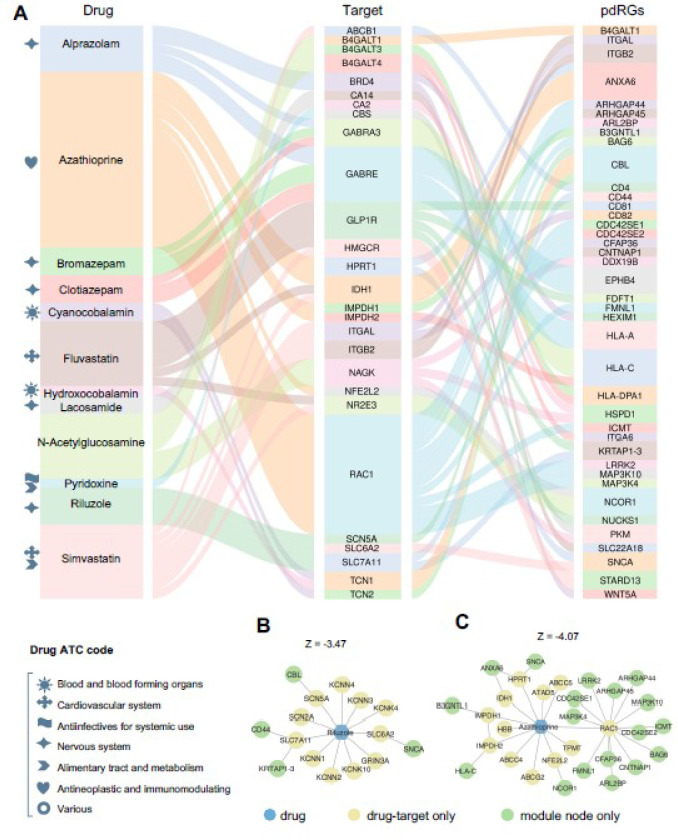
Repurposable drug candidates for PD treatment based on network proximity method. (A) Sankey plot illustrating targets and pdRGs for prioritized twelve PD drugs (**Table S7**). The grey symbols in front of the drug name characterize the anatomical therapeutic chemical code (ATC). (B-C) Mechanism of actions (MOAs) of two predicted drugs, including (A) riluzole and (B) azathioprine.

Within the well-established drug-target network [58], we found that pdRG-encoded proteins were significantly enriched as druggable targets (41 proteins [~23%, *p* = 0.009], Figure 3B). We next prioritized potential repurposable drugs by targeting the protein products of 175 pdRGs (*cf.* Methods). Here, we calculated network-based proximity scores between pdRGs-encoded proteins and the drug-target network with the human protein-protein interactome network [58]. Using criteria of (1) a stronger network proximity (Z < −3) and (2) existing preclinical and experimental evidence of drugs related to PD, we prioritized 12 U.S. Food and Drug Administration (FDA)-approved candidate drugs (Figure 6 and Supplementary **Table S7**), including simvastatin, fluvastatin, n-acetylglucosamine, azathioprine, riluzole, clotiazepam, cyanocobalamin, bromazepam, hydroxocobalamin, lacosamide, pyridoxine, and alprazolam. Figure 6A illustrates connections among drugs, targets, and pdRGs (connected by protein interactions with drug targets).

According to the first level of the anatomical therapeutic chemical (ATC) code, these top network proximity-ranked drugs group into seven pharmacological categories. For example, two drugs of simvastatin and fluvastatin (i.e., statins), are categorized in the cardiovascular system and four drugs of riluzole, alprazolam, bromazepam, and clotiazepam in the nervous system. Riluzole is an approved medicine by the FDA for amyotrophic lateral sclerosis (ALS) that reduces glutamate release in the brain [71]. Network-based mechanism-of-action (MOA) analysis showed that riluzole may interact with four targets (*CBL*, *CD44*, *KRTAP*1–1 and *SNCA*) encoded by pdRGs (Figure 4B). Azathioprine, another top-predicted immunosuppressant, is currently in a phase II PD clinical trial (AZA-PD) [72] and drug-target network analysis reveals that azathioprine may target multiple protein products of pdRGs, including *LRRK2*, *BAG6*, *SNCA*, *HLA-C* (Figure 4C). In summary, these network proximity analyses showed that pdRGs offer potential druggable targets to identify candidate repurposable drugs (i.e., simvastatin) for PD.

### Simvastatin is associated with reduced PD incidence in real-world data

**Figure 7:**
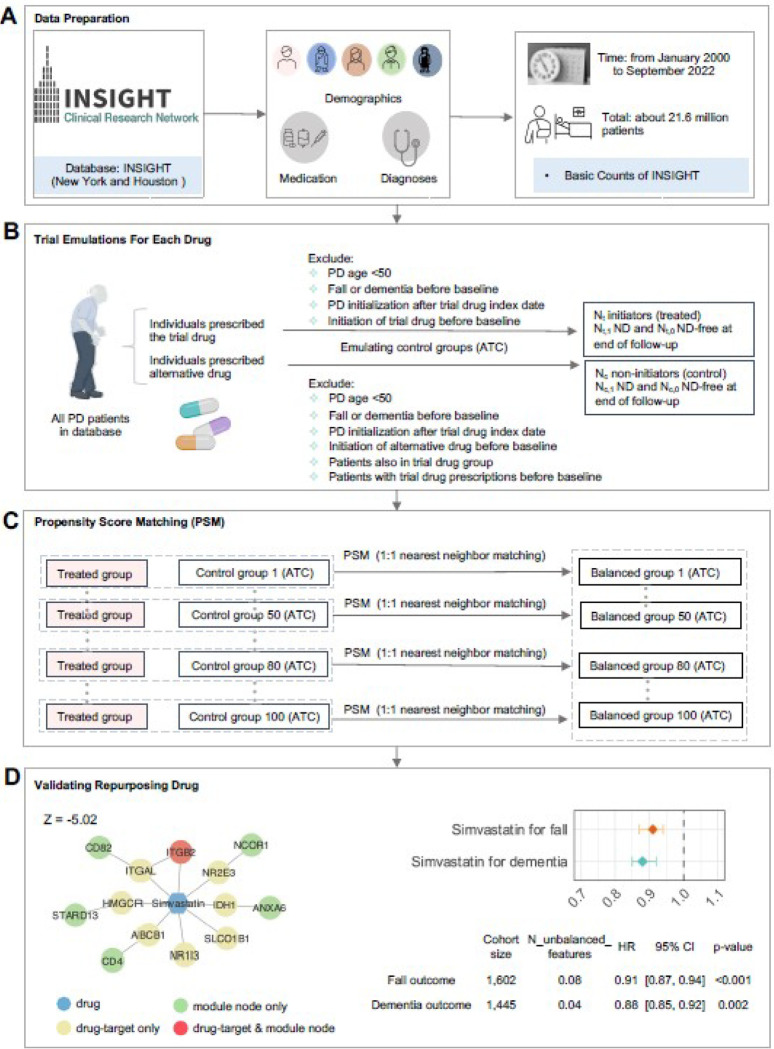
Longitudinal patient data validation for potential PD drug candidates. (A) Basic characteristics of INSIGHT database. (B) Trial emulation for each drug with consideration of two kinds of PD outcomes, i.e., fall and dementia. (C) Perform propensity score matching (PSM) to control confounding factors and build balanced trials. (D) MOAs of simvastatin, along with the forest plot of the hazard ratio (HR).

We next selected candidate drugs to test drug-disease outcome using subject matter expertise based on a combination of factors: (i) strength of the network proximity score (Supplementary **Table 7**); (ii) availability of sufficient patient data for meaningful evaluation from the patient database; and (iii) existing literature-reported preclinical and clinical evidence related to PD. Applying all of these criteria resulted in *Simvastatin (Z = −5.02)* emerging as the best candidate drug. We next sought to further evaluate the simvastatin-PD outcome using the large real-world patient EHR data from the INSIGHT dataset (Figure 7 and Supplementary **Tables S8–10**) with 5,703 fall-diagnosed and 13,244 dementia-diagnosed PD patients. To adjust for confounding factors, we performed a propensity score matching (PSM) analysis using logistic regression with ridge penalty (267 covariates). Nearest-neighbor matching (1:1) was applied to construct balanced emulated trials for simvastatin cohorts. We found that simvastatin was significantly associated with reduced incidence of PD using the Cox proportional hazard model (Figure 7D and Supplementary **Table S10**). Specifically, simvastatin was significantly associated with 12% (95% confidence interval [95% CI: 0.85–0.92, *p* = 0.002]) reduced PD-related dementia. In addition, simvastatin was significantly associated with 9% (95% CI: [0.87–0.94, *p* < 0.001]) reduced risk of fall (secondary outcome). Simvastatin is used to reduce lipid levels by blocking the enzyme 3-hydroxy-3-methylglutaryl-CoA (HMG-CoA) reductase, thereby decreasing low-density lipoprotein (LDL) cholesterol synthesis. Drug-target network analysis shows that simvastatin directly targets one pdRG-encoded protein *ITGB2* (integrin subunit beta 2, previously known as *CD18*) (Figure 7D). *ITGB2* has been reported to have functional roles in neurodegenerative diseases, including PD [66, 73]. Furthermore, simvastatin also targets the PPI network neighbors of five other proteins encoded by pdRGs of *CD4, STARD13, CD82, NCOR1* and *ANXA6*, revealing potential mechanism-of-actions (MOAs) for treatment of PD. However, experimental observations are warranted to determine the MOAs of simvastatin’s beneficial effects on PD in the future.

## Discussion

Parkinson’s Disease (PD) is a neurological disorder characterized by the classical motor features of parkinsonism associated with loss of dopaminergic neurons in the substantia nigra [74, 75]. Recent studies have also shown that PD is governed by network-associated molecular determinants (termed disease module) of common intermediate endophenotypes [5, 76]. While approaching PD with a direct single-target approach has successfully developed symptomatic therapies, this approach has not achieved disease-modifying or neuroprotective therapies [77]. Discovery of therapeutic approaches by specifically targeting genetics-informed risk genes is essential for developing disease-modifying treatments for PD [76, 78]. In this study, we presented a network-based deep learning approach from unique integration of PD GWAS loci and human brain-specific functional genomics profiles. The fundamental premise of our network-based framework was that likely risk genes of PD exhibit distinct functional characteristics compared to non-risk genes and, therefore, can be distinguished by their aggregated brain-specific genome-wide quantitative trait loci (x-QTLs) features under the human protein-protein interactome network. In totally, we identified 175 putative risk genes for PD (termed pdRGs) via unique integration of existing PD GWAS loci into human brain-specific eQTL, pQTL, sQTL, meQTL, and haQTL. We demonstrated that these predicted PD-associated genes were significantly enriched in various PD-related pathobiological pathways: (1) enriched in parkin-dependent substrates and alpha-synuclein (*α*-syn) modifiers, (2) highly expressed in human brain substantia nigra (SN) region; (3) more likely differentially expressed in human PD brains in cell type-specific manners, including DA neurons; and (4) significantly enriched in literature-reported PD pathways and known drug targets.

Integrated analysis of single nuclei chromatin accessibility (snATAC-seq) or multiome data (simultaneously scRNA-seq and scATAC-seq for same nuclei) facilitates the investigation of genetic risk loci through cis-regulatory elements (cREs) at the cell type resolution. A total of 656 target genes of dysregulated cREs and PD GWAS-SNPs were identified in a cell type–specific manner by lee et al. (PD vs. HC) [79], including nine identified pdRGs of *TRPM7*, *NEIL2*, *MAPT*, *TNK2*, *SNCA*, *NAA25*, *TAPBP*, *KDM3A*, *EFNA1*. Specifically, four pdRGs were observed in DA neurons, including *MAPT* (cRE ID: chr17:43970936–43973745, GWAS significant peak), *TRPM7*, *NEIL2*, *TNK2*. As for MG, also four pdRGs were found to be related to dysregulated cREs, including *SNCA* (cRE ID: chr4:90750339–90751275, GWAS significant peak), *TRPM7*, *TAPBP*, and *KDM3A*. Notably, *MAPT* was observed in multiple cell types (DA neurons, GABA neurons, ODC, OPC, astrocytes), as well as *TRPM7* (DA neurons, GABA neurons, ODC, OPC, astrocytes and pericytes).

Using network proximity-based prediction, we identified 12 candidate drugs for potential prevention or treatment of PD. Among the predicted drug candidates, simvastatin and fluvastatin belong to the statin class of lipid-lowering agents. Many studies suggest that statins play protective roles in vascular and degenerative disorders through inflammatory and lysosomal signaling pathways. A recent clinical trial (covering 2,841 older adults) found that statin use may reduce the risk of PD [80], and a meta-analysis (containing 3,845,303 patients, 8 case-control and 9 cohort studies) also provided evidence for a protective effect of statins in PD, especially atorvastatin [81]. Beyond statins, riluzole, and azathioprine, we also highlighted eight other drugs with computational evidence of the potential to slow PD progression (Figure 6 and Supplementary **Figure S2**). Three anti-anxiety drugs (clotiazepam, alprazolam, and bromazepam) were highly ranked and shared similar MOAs. Notably, anxiety is a common non-motor symptom of PD that is experienced by up to 40% of PD patients and a randomized controlled clinical study found that bromazepam is effective for anxiety in PD [82]. Vitamin B12 (VB12) supplements, including hydroxocobalamin and cyanocobalamin, were also identified, and many studies have reported that VB12 level is significantly lower in PD than in healthy people [83, 84]. Interestingly, VB12 modulates *LRRK2* kinase activity through allosteric regulation to protect neurons and both VB12 supplements shared similar MOAs. Specifically, they interact with proteins encoded by pdRG *CBL* (Cbl proto-oncogene), one of the enzymes required for targeting substrates for degradation by the proteasome [85]. We also identified pyridoxine, which has been shown to promote glutathione synthesis to eliminate oxidative damage to protect DA neurons in a mouse model [86], and N-acetyl-glucosamine (O-GlcNAc), which is a sugar-like molecule that can attach to *α*-syn to affect *α*-syn clumping [87]. Lastly, lacosamide also holds promise as a neuroprotective agent in a PD rat model through reducing neuroinflammation and oxidative stress [88].

We acknowledge some limitations in the current study. For example, we integrated only two PD-related GWAS studies [7, 19]. The latest GWAS data [89] with higher population size should be integrated in the future study. Additionally, to keep more risk variants for downstream mapping and pdRGs prediction, we applied the less stringent *p*-value thresholds (*p* < 5×10^−5^, leading to 124 xQTL-regulated genes) instead of genome-wide significance (*p* < 1×10^−8^, leading to 70 QTL-regulated genes) [90–92]. Furthermore, the limited number of brain-specific functional genomics datasets, such as sQTLs, pQTLs, and haQTLs, may cause the overall low AUROC in our framework (Figure 3A). Moreover, some evidence supported the involvement of periphery in PD, such as pathological process originating in the gut [93, 94]. Therefore, beyond brain-specific QTL data, the contribution of other types of QTL data (i.e., colon-specific QTLs, small Intestine - terminal Ileum QTLs) [39] need to be explored in the future. Cell type-specific QTLs may also have great potential to improve model accuracy further [95, 96].

Using large real-world EHR database, we demonstrated that simvastatin was significantly associated with reduce risk of dementia and fall (Figure 7), offering potential treatment approaches for PD. Yet, the EHR-based observation also has certain limitations. First, since the real drug administration data are unavailable, we can only consider whether patients were prescribed the drugs. Although patients may generally adhere to prescriptions, this is not always the case. Second, pharmacoepidemiologic studies may be biased due to unavailable confounding factors (i.e., ethnicity, education level, socioeconomic status). Therefore, it is difficult to build causal relationships between drug use and clinical benefits via EHR observation. Most importantly, multiple proposed drugs failed due to the unsatisfactory cohort size using INSIGHT database. A larger-scale EHR data might help validate more drugs and generate more robust results.

Computational strategies are an important evolving approach to nominating candidate drugs for treatment of neurodegenerative disorders including PD. Once identified as promising therapies, these candidates can be further interrogated through cellular and animal models to generate support for the likelihood of clinical efficacy and to determine if they should be advanced for assessment in clinical trials.

## Data Availability

PD GWAS summary results can be download from GWAS catalog (study IDs: GCST009325, GCST007780)[7, 19]. The brain-specific eQTL data are available through the AMP-AD Knowledge Portal (syn25398075) [20] and MetaBrain website [21] (https://metabrain.nl/). And pQTL data can also be obtained on the AMP-AD Knowledge Portal (syn24172458 [22] and syn23627957 [23]). The full summary statistics from the sQTL are available at https://yanglab.westlake.edu.cn/data/brainmeta/cis_sqtl/ [24]. And meQTL and haQTL are both collected from Brain xQTLServe (http://mostafavilab.stat.ubc.ca/xQTLServe) [25]. The snRNA-seq dataset for PD/LBD were download from the Gene Expression Omnibus database under accession of GSE178265[35]. Predicted pdRGs and prioritized drugs were listed in supplementary files. The codes for the proposed genetic-supported risk gene prediction model, snRNA-seq analysis pipeline are available at https://github.com/ChengF-Lab/PDNetworks.

## References

[1] Feigin VL, Nichols E, Alam T, Bannick MS, Beghi E, Blake N, Culpepper WJ, Dorsey ER, Elbaz A, Ellenbogen RG, : Global, regional, and national burden of neurological disorders, 1990–2016: a systematic analysis for the Global Burden of Disease Study 2016. The Lancet Neurology 2019, 18:459–480. 30879893 10.1016/S1474-4422(18)30499-X PMC6459001

[2] Moustafa AA, Chakravarthy S, Phillips JR, Gupta A, Keri S, Polner B, Frank MJ, Jahanshahi M: Motor symptoms in Parkinson’s disease: A unified framework. Neuroscience & Biobehavioral Reviews 2016, 68:727–740. 27422450 10.1016/j.neubiorev.2016.07.010

[3] Cilia R, Cereda E, Akpalu A, Sarfo FS, Cham M, Laryea R, Obese V, Oppon K, Del Sorbo F, Bonvegna S, : Natural history of motor symptoms in Parkinson’s disease and the long-duration response to levodopa. Brain 2020, 143:2490–2501. 32844196 10.1093/brain/awaa181 PMC7566883

[4] Tibar H, El Bayad K, Bouhouche A, Ait Ben Haddou EH, Benomar A, Yahyaoui M, Benazzouz A, Regragui W: Non-Motor Symptoms of Parkinson’s Disease and Their Impact on Quality of Life in a Cohort of Moroccan Patients. Frontiers in Neurology 2018, 9. 10.3389/fneur.2018.00170 PMC5893866 29670566

[5] Armstrong MJ, Okun MS: Diagnosis and Treatment of Parkinson Disease: A Review. JAMA 2020, 323:548–560. 32044947 10.1001/jama.2019.22360

[6] Poewe W, Seppi K, Marini K, Mahlknecht P: New hopes for disease modification in Parkinson’s Disease. Neuropharmacology 2020, 171:108085. 32298705 10.1016/j.neuropharm.2020.108085

[7] Nalls MA, Blauwendraat C, Vallerga CL, Heilbron K, Bandres-Ciga S, Chang D, Tan M, Kia DA, Noyce AJ, Xue A, : Identification of novel risk loci, causal insights, and heritable risk for Parkinson’s disease: a meta-analysis of genome-wide association studies. Lancet Neurol 2019, 18:1091–1102. 31701892 10.1016/S1474-4422(19)30320-5 PMC8422160

[8] Cano-Gamez E, Trynka G: From GWAS to Function: Using Functional Genomics to Identify the Mechanisms Underlying Complex Diseases. Frontiers in Genetics 2020, 11. 10.3389/fgene.2020.00424 PMC7237642 32477401

[9] Visscher PM, Wray NR, Zhang Q, Sklar P, McCarthy MI, Brown MA, Yang J: 10 Years of GWAS Discovery: Biology, Function, and Translation. Am J Hum Genet 2017, 101:5–22. 28686856 10.1016/j.ajhg.2017.06.005 PMC5501872

[10] Xu J, Zhang P, Huang Y, Zhou Y, Hou Y, Bekris L, Lathia J, Chiang C-W, Li L, Pieper A, : Multimodal single-cell/nucleus RNA sequencing data analysis uncovers molecular networks between disease-associated microglia and astrocytes with implications for drug repurposing in Alzheimer’s disease. Genome Research 2021, 31:gr.272484.272120. 10.1101/gr.272484.120 PMC8494225 33627474

[11] Fang J, Zhang P, Zhou Y, Chiang C-W, Tan J, Hou Y, Stauffer S, Li L, Pieper AA, Cummings J, Cheng F: Endophenotype-based in silico network medicine discovery combined with insurance record data mining identifies sildenafil as a candidate drug for Alzheimer’s disease. Nature Aging 2021, 1:1175–1188. 35572351 10.1038/s43587-021-00138-z PMC9097949

[12] Swarup V, Hinz FI, Rexach JE, Noguchi K-i, Toyoshiba H, Oda A, Hirai K, Sarkar A, Seyfried NT, Cheng C, : Identification of evolutionarily conserved gene networks mediating neurodegenerative dementia. Nature Medicine 2019, 25:152–164. 10.1038/s41591-018-0223-3 PMC6602064 30510257

[13] Fang J, Pieper AA, Nussinov R, Lee G, Bekris L, Leverenz JB, Cummings J, Cheng F: Harnessing endophenotypes and network medicine for Alzheimer’s drug repurposing. Medicinal Research Reviews 2020, 40:2386–2426. 32656864 10.1002/med.21709 PMC7561446

[14] Zhou Y, Xu J, Hou Y, Leverenz JB, Kallianpur A, Mehra R, Liu Y, Yu H, Pieper AA, Jehi L, Cheng F: Network medicine links SARS-CoV-2/COVID-19 infection to brain microvascular injury and neuroinflammation in dementia-like cognitive impairment. Alzheimer’s Research & Therapy 2021, 13:110. 10.1186/s13195-021-00850-3 PMC8189279 34108016

[15] Cheng F, Lu W, Liu C, Fang J, Hou Y, Handy DE, Wang R, Zhao Y, Yang Y, Huang J, : A genome-wide positioning systems network algorithm for in silico drug repurposing. Nature Communications 2019, 10:3476. 10.1038/s41467-019-10744-6 PMC6677722 31375661

[16] Xu J, Mao C, Hou Y, Luo Y, Binder JL, Zhou Y, Bekris LM, Shin J, Hu M, Wang F, : Interpretable deep learning translation of GWAS and multi-omics findings to identify pathobiology and drug repurposing in Alzheimer’s disease. Cell Reports 2022, 41:111717. 36450252 10.1016/j.celrep.2022.111717 PMC9837836

[17] Fang J, Zhang P, Wang Q, Chiang C-W, Zhou Y, Hou Y, Xu J, Chen R, Zhang B, Lewis SJ, : Artificial intelligence framework identifies candidate targets for drug repurposing in Alzheimer’s disease. Alzheimer’s Research & Therapy 2022, 14:7. 10.1186/s13195-021-00951-z PMC8751379 35012639

[18] Cheng F, Wang F, Tang J, Zhou Y, Fu Z, Zhang P, Haines JL, Leverenz JB, Gan L, Hu J, : Artificial intelligence and open science in discovery of disease-modifying medicines for Alzheimer’s disease. Cell Reports Medicine 2024, 5:101379. 38382465 10.1016/j.xcrm.2023.101379 PMC10897520

[19] Blauwendraat C, Heilbron K, Vallerga CL, Bandres-Ciga S, von Coelln R, Pihlstrøm L, Simón-Sánchez J, Schulte C, Sharma M, Krohn L, : Parkinson’s disease age at onset genome-wide association study: Defining heritability, genetic loci, and α-synuclein mechanisms. Movement Disorders 2019, 34:866–875. 30957308 10.1002/mds.27659 PMC6579628

[20] Sieberts SK, Perumal TM, Carrasquillo MM, Allen M, Reddy JS, Hoffman GE, Dang KK, Calley J, Ebert PJ, Eddy J, : Large eQTL meta-analysis reveals differing patterns between cerebral cortical and cerebellar brain regions. Sci Data 2020, 7:340. 33046718 10.1038/s41597-020-00642-8 PMC7550587

[21] de Klein N, Tsai EA, Vochteloo M, Baird D, Huang Y, Chen C-Y, van Dam S, Oelen R, Deelen P, Bakker OB, : Brain expression quantitative trait locus and network analyses reveal downstream effects and putative drivers for brain-related diseases. Nature Genetics 2023, 55:377–388. 36823318 10.1038/s41588-023-01300-6 PMC10011140

[22] Robins C, Liu Y, Fan W, Duong DM, Meigs J, Harerimana NV, Gerasimov ES, Dammer EB, Cutler DJ, Beach TG, : Genetic control of the human brain proteome. The American Journal of Human Genetics 2021, 108:400–410. 33571421 10.1016/j.ajhg.2021.01.012 PMC8008492

[23] Wingo AP, Liu Y, Gerasimov ES, Gockley J, Logsdon BA, Duong DM, Dammer EB, Robins C, Beach TG, Reiman EM, : Integrating human brain proteomes with genome-wide association data implicates new proteins in Alzheimer’s disease pathogenesis. Nature Genetics 2021, 53:143–146. 33510477 10.1038/s41588-020-00773-z PMC8130821

[24] Qi T, Wu Y, Fang H, Zhang F, Liu S, Zeng J, Yang J: Genetic control of RNA splicing and its distinct role in complex trait variation. Nature Genetics 2022, 54:1355–1363. 35982161 10.1038/s41588-022-01154-4 PMC9470536

[25] Ng B, White CC, Klein H-U, Sieberts SK, McCabe C, Patrick E, Xu J, Yu L, Gaiteri C, Bennett DA, : An xQTL map integrates the genetic architecture of the human brain’s transcriptome and epigenome. Nature Neuroscience 2017, 20:1418–1426. 28869584 10.1038/nn.4632 PMC5785926

[26] Quinlan AR, Hall IM: BEDTools: a flexible suite of utilities for comparing genomic features. Bioinformatics 2010, 26:841–842. 20110278 10.1093/bioinformatics/btq033 PMC2832824

[27] Cui Y, Chen X, Luo H, Fan Z, Luo J, He S, Yue H, Zhang P, Chen R: BioCircos.js: an interactive Circos JavaScript library for biological data visualization on web applications. Bioinformatics 2016, 32:1740–1742. 26819473 10.1093/bioinformatics/btw041

[28] Pruim RJ, Welch RP, Sanna S, Teslovich TM, Chines PS, Gliedt TP, Boehnke M, Abecasis GR, Willer CJ: LocusZoom: regional visualization of genome-wide association scan results. Bioinformatics 2010, 26:2336–2337. 20634204 10.1093/bioinformatics/btq419 PMC2935401

[29] Yang JJ, Grissa D, Lambert CG, Bologa CG, Mathias SL, Waller A, Wild DJ, Jensen LJ, Oprea TI: TIGA: target illumination GWAS analytics. Bioinformatics 2021, 37:3865–3873. 34086846 10.1093/bioinformatics/btab427 PMC11025677

[30] Luck K, Kim D-K, Lambourne L, Spirohn K, Begg BE, Bian W, Brignall R, Cafarelli T, Campos-Laborie FJ, Charloteaux B, : A reference map of the human binary protein interactome. Nature 2020, 580:402–408. 32296183 10.1038/s41586-020-2188-x PMC7169983

[31] Fazekas D, Koltai M, Türei D, Módos D, Pálfy M, Dúl Z, Zsákai L, Szalay-Bekő M, Lenti K, Farkas IJ, : SignaLink 2 – a signaling pathway resource with multi-layered regulatory networks. BMC Systems Biology 2013, 7:7. 23331499 10.1186/1752-0509-7-7 PMC3599410

[32] Meyer MJ, Das J, Wang X, Yu H: INstruct: a database of high-quality 3D structurally resolved protein interactome networks. Bioinformatics 2013, 29:1577–1579. 23599502 10.1093/bioinformatics/btt181 PMC3673217

[33] Huttlin EL, Ting L, Bruckner RJ, Gebreab F, Gygi MP, Szpyt J, Tam S, Zarraga G, Colby G, Baltier K, : The BioPlex Network: A Systematic Exploration of the Human Interactome. Cell 2015, 162:425–440. 26186194 10.1016/j.cell.2015.06.043 PMC4617211

[34] Xu K, Li C, Tian Y, Sonobe T, Kawarabayashi K-i, Jegelka S: Representation learning on graphs with jumping knowledge networks. In International conference on machine learning. PMLR; 2018: 5453–5462.

[35] Kamath T, Abdulraouf A, Burris SJ, Langlieb J, Gazestani V, Nadaf NM, Balderrama K, Vanderburg C, Macosko EZ: Single-cell genomic profiling of human dopamine neurons identifies a population that selectively degenerates in Parkinson’s disease. Nature Neuroscience 2022, 25:588–595. 35513515 10.1038/s41593-022-01061-1 PMC9076534

[36] McGinnis CS, Murrow LM, Gartner ZJ: DoubletFinder: Doublet Detection in Single-Cell RNA Sequencing Data Using Artificial Nearest Neighbors. Cell Systems 2019, 8:329–337.e324. 30954475 10.1016/j.cels.2019.03.003 PMC6853612

[37] Korsunsky I, Millard N, Fan J, Slowikowski K, Zhang F, Wei K, Baglaenko Y, Brenner M, Loh P-r, Raychaudhuri S: Fast, sensitive and accurate integration of single-cell data with Harmony. Nature Methods 2019, 16:1289–1296. 31740819 10.1038/s41592-019-0619-0 PMC6884693

[38] Stuart T, Butler A, Hoffman P, Hafemeister C, Papalexi E, Mauck WM III, Hao Y, Stoeckius M, Smibert P, Satija R: Comprehensive Integration of Single-Cell Data. Cell 2019, 177:1888–1902.e1821. 31178118 10.1016/j.cell.2019.05.031 PMC6687398

[39] Lonsdale J, Thomas J, Salvatore M, Phillips R, Lo E, Shad S, Hasz R, Walters G, Garcia F, Young N, : The Genotype-Tissue Expression (GTEx) project. Nature Genetics 2013, 45:580–585. 23715323 10.1038/ng.2653 PMC4010069

[40] Shannon P, Markiel A, Ozier O, Baliga NS, Wang JT, Ramage D, Amin N, Schwikowski B, Ideker T: Cytoscape: A Software Environment for Integrated Models of Biomolecular Interaction Networks. Genome Research 2003, 13:2498–2504. 14597658 10.1101/gr.1239303 PMC403769

[41] Cheng F, Desai RJ, Handy DE, Wang R, Schneeweiss S, Barabási A-L, Loscalzo J: Network-based approach to prediction and population-based validation of in silico drug repurposing. Nature Communications 2018, 9:2691. 10.1038/s41467-018-05116-5 PMC6043492 30002366

[42] Ozery-Flato M, Goldschmidt Y, Shaham O, Ravid S, Yanover C: Framework for identifying drug repurposing candidates from observational healthcare data. JAMIA Open 2020, 3:536–544. 33623890 10.1093/jamiaopen/ooaa048 PMC7886555

[43] Zang C, Zhang H, Xu J, Zhang H, Fouladvand S, Havaldar S, Cheng F, Chen K, Chen Y, Glicksberg BS, : High-Throughput Clinical Trial Emulation with Real World Data and Machine Learning: A Case Study of Drug Repurposing for Alzheimer’s Disease. medRxiv 2022:2022.2001.2031.22270132.

[44] Allan V, Ramagopalan SV, Mardekian J, Jenkins A, Li X, Pan X, Luo X: Propensity score matching and inverse probability of treatment weighting to address confounding by indication in comparative effectiveness research of oral anticoagulants. Journal of Comparative Effectiveness Research 2020, 9:603–614. 32186922 10.2217/cer-2020-0013

[45] Austin PC: Balance diagnostics for comparing the distribution of baseline covariates between treatment groups in propensity-score matched samples. Statistics in Medicine 2009, 28:3083–3107. 19757444 10.1002/sim.3697 PMC3472075

[46] Liu R, Wei L, Zhang P: A deep learning framework for drug repurposing via emulating clinical trials on real-world patient data. Nature Machine Intelligence 2021, 3:68–75. 10.1038/s42256-020-00276-w PMC9119409 35603127

[47] Lin DY, Wei LJ: The Robust Inference for the Cox Proportional Hazards Model. Journal of the American Statistical Association 1989, 84:1074–1078.

[48] DiCiccio TJ, Efron B: Bootstrap confidence intervals. Statistical Science 1996, 11:189–228, 140.

[49] Kline A, Luo Y: PsmPy: A Package for Retrospective Cohort Matching in Python. In 2022 44th Annual International Conference of the IEEE Engineering in Medicine & Biology Society (EMBC); 11–15 July 2022. 2022: 1354–1357. 10.1109/EMBC48229.2022.9871333 36086543

[50] Davidson-Pilon: lifelines: survival analysis in Python. Journal of Open Source Software 2019, 4:1317.

[51] Diaz-Ortiz ME, Seo Y, Posavi M, Carceles Cordon M, Clark E, Jain N, Charan R, Gallagher MD, Unger TL, Amari N, : GPNMB confers risk for Parkinson’s disease through interaction with α-synuclein. Science 2022, 377:eabk0637. 35981040 10.1126/science.abk0637 PMC9870036

[52] Boeve BF, Hutton M: Refining Frontotemporal Dementia With Parkinsonism Linked to Chromosome 17: Introducing FTDP-17 (MAPT) and FTDP-17 (PGRN). Archives of Neurology 2008, 65:460–464. 18413467 10.1001/archneur.65.4.460 PMC2746630

[53] Guo P, Gong W, Li Y, Liu L, Yan R, Wang Y, Zhang Y, Yuan Z: Pinpointing novel risk loci for Lewy body dementia and the shared genetic etiology with Alzheimer’s disease and Parkinson’s disease: a large-scale multi-trait association analysis. BMC Medicine 2022, 20:214. 35729600 10.1186/s12916-022-02404-2 PMC9214990

[54] Sollis E, Mosaku A, Abid A, Buniello A, Cerezo M, Gil L, Groza T, Güneş O, Hall P, Hayhurst J, : The NHGRI-EBI GWAS Catalog: knowledgebase and deposition resource. Nucleic Acids Research 2022, 51:D977–D985. 10.1093/nar/gkac1010 PMC9825413 36350656

[55] Sarraf SA, Raman M, Guarani-Pereira V, Sowa ME, Huttlin EL, Gygi SP, Harper JW: Landscape of the PARKIN-dependent ubiquitylome in response to mitochondrial depolarization. Nature 2013, 496:372–376. 23503661 10.1038/nature12043 PMC3641819

[56] Chung CY, Khurana V, Yi S, Sahni N, Loh KH, Auluck PK, Baru V, Udeshi ND, Freyzon Y, Carr SA, : In Situ Peroxidase Labeling and Mass-Spectrometry Connects Alpha-Synuclein Directly to Endocytic Trafficking and mRNA Metabolism in Neurons. Cell Systems 2017, 4:242–250.e244. 28131823 10.1016/j.cels.2017.01.002 PMC5578869

[57] Lu L, Neff F, Fischer DA, Henze C, Hirsch EC, Oertel WH, Schlegel J, Hartmann A: Regional vulnerability of mesencephalic dopaminergic neurons prone to degenerate in Parkinson’s disease: A post-mortem study in human control subjects. Neurobiology of Disease 2006, 23:409–421. 16753304 10.1016/j.nbd.2006.04.002

[58] Zhou Y, Xu J, Hou Y, Bekris L, Leverenz JB, Pieper AA, Cummings J, Cheng F: The Alzheimer’s Cell Atlas (TACA): A single-cell molecular map for translational therapeutics accelerator in Alzheimer’s disease. Alzheimer’s & Dementia: Translational Research & Clinical Interventions 2022, 8:e12350. 10.1002/trc2.12350 PMC9558163 36254161

[59] Kamienieva I, Duszyński J, Szczepanowska J: Multitasking guardian of mitochondrial quality: Parkin function and Parkinson’s disease. Translational Neurodegeneration 2021, 10:5. 33468256 10.1186/s40035-020-00229-8 PMC7816312

[60] Pickrell Alicia M, Youle Richard J: The Roles of PINK1, Parkin, and Mitochondrial Fidelity in Parkinson’s Disease. Neuron 2015, 85:257–273. 25611507 10.1016/j.neuron.2014.12.007 PMC4764997

[61] Bloem BR, Okun MS, Klein C: Parkinson’s disease. The Lancet 2021, 397:2284–2303. 10.1016/S0140-6736(21)00218-X 33848468

[62] Kam T-I, Hinkle JT, Dawson TM, Dawson VL: Microglia and astrocyte dysfunction in parkinson’s disease. Neurobiology of Disease 2020, 144:105028. 32736085 10.1016/j.nbd.2020.105028 PMC7484088

[63] Wood H: α-Synuclein-activated microglia are implicated in PD pathogenesis. Nature Reviews Neurology 2022, 18:188–188. 10.1038/s41582-022-00631-y 35165429

[64] Bartels T, De Schepper S, Hong S: Microglia modulate neurodegeneration in Alzheimer’s and Parkinson’s diseases. Science 2020, 370:66–69. 33004513 10.1126/science.abb8587

[65] Yao S, Zhang X, Zou S-C, Zhu Y, Li B, Kuang W-P, Guo Y, Li X-S, Li L, Wang X-Y: A transcriptome-wide association study identifies susceptibility genes for Parkinson’s disease. npj Parkinson’s Disease 2021, 7:79. 10.1038/s41531-021-00221-7 PMC8429416 34504106

[66] Phillips B, Western D, Wang L, Timsina J, Sun Y, Gorijala P, Yang C, Do A, Nykänen N-P, Alvarez I, : Proteome wide association studies of LRRK2 variants identify novel causal and druggable proteins for Parkinson’s disease. npj Parkinson’s Disease 2023, 9:107. 10.1038/s41531-023-00555-4 PMC10329646 37422510

[67] Joshi N, Raveendran A, Nagotu S: Chaperones and Proteostasis: Role in Parkinson’s Disease. Diseases 2020, 8:24. 32580484 10.3390/diseases8020024 PMC7349525

[68] Kim YJ, Kim K, Lee H, Jeon J, Lee J, Yoon J: The Protein-Protein Interaction Network of Hereditary Parkinsonism Genes Is a Hierarchical Scale-Free Network. Yonsei Med J 2022, 63:724–734. 35914754 10.3349/ymj.2022.63.8.724 PMC9344267

[69] Ham SJ, Lee D, Xu WJ, Cho E, Choi S, Min S, Park S, Chung J: Loss of UCHL1 rescues the defects related to Parkinson’s disease by suppressing glycolysis. Science Advances 2021, 7:eabg4574. 34244144 10.1126/sciadv.abg4574 PMC8270484

[70] Williams GP, Schonhoff AM, Jurkuvenaite A, Gallups NJ, Standaert DG, Harms AS: CD4 T cells mediate brain inflammation and neurodegeneration in a mouse model of Parkinson’s disease. Brain 2021, 144:2047–2059. 33704423 10.1093/brain/awab103 PMC8370411

[71] Carbone M, Duty S, Rattray M: Riluzole neuroprotection in a parkinson’s disease model involves suppression of reactive astrocytosis but not GLT-1 regulation. BMC Neuroscience 2012, 13:38. 22480308 10.1186/1471-2202-13-38 PMC3349538

[72] Greenland JC, Cutting E, Kadyan S, Bond S, Chhabra A, Williams-Gray CH: Azathioprine immunosuppression and disease modification in Parkinson’s disease (AZA-PD): a randomised double-blind placebo-controlled phase II trial protocol. BMJ Open 2020, 10:e040527. 10.1136/bmjopen-2020-040527 PMC7684836 33234645

[73] Capelle CM, Ciré S, Hedin F, Hansen M, Pavelka L, Grzyb K, Kyriakis D, Hunewald O, Konstantinou M, Revets D, : Early-to-mid stage idiopathic Parkinson’s disease shows enhanced cytotoxicity and differentiation in CD8 T-cells in females. Nature Communications 2023, 14:7461. 10.1038/s41467-023-43053-0 PMC10662447 37985656

[74] Carceles-Cordon M, Weintraub D, Chen-Plotkin AS: Cognitive heterogeneity in Parkinson’s disease: A mechanistic view. *Neuron* 2023, 111:1531–1546. 37028431 10.1016/j.neuron.2023.03.021 PMC10198897

[75] Kalia LV, Lang AE: Parkinson’s disease. The Lancet 2015, 386:896–912. 10.1016/S0140-6736(14)61393-3 25904081

[76] Elkouzi A, Vedam-Mai V, Eisinger RS, Okun MS: Emerging therapies in Parkinson disease — repurposed drugs and new approaches. Nature Reviews Neurology 2019, 15:204–223. 30867588 10.1038/s41582-019-0155-7 PMC7758837

[77] Espay AJ, Brundin P, Lang AE: Precision medicine for disease modification in Parkinson disease. Nature Reviews Neurology 2017, 13:119–126. 28106064 10.1038/nrneurol.2016.196

[78] Chen Y, Cai X, Xu R: Combining Human Disease Genetics and Mouse Model Phenotypes towards Drug Repositioning for Parkinson’s disease. AMIA Annu Symp Proc 2015, 2015:1851–1860. 26958284 PMC4765695

[79] Lee AJ, Kim C, Park S, Joo J, Choi B, Yang D, Jun K, Eom J, Lee S-J, Chung SJ, : Characterization of altered molecular mechanisms in Parkinson’s disease through cell type–resolved multiomics analyses. Science Advances 2023, 9:eabo2467. 37058563 10.1126/sciadv.abo2467 PMC10104466

[80] Oveisgharan S, Yu L, Barnes LL, Agrawal S, Schneider JA, Bennett DA, Buchman AS: Association of Statins With Cerebral Atherosclerosis and Incident Parkinsonism in Older Adults. Neurology 2022, 98:e1976–e1984. 35321928 10.1212/WNL.0000000000200182 PMC9141626

[81] Yan J, Qiao L, Tian J, Liu A, Wu J, Huang J, Shen M, Lai X: Effect of statins on Parkinson’s disease: A systematic review and meta-analysis. Medicine 2019, 98. 10.1097/MD.0000000000014852 PMC6709163 30896628

[82] Chen JJ, Marsh L: Anxiety in Parkinson’s disease: identification and management. Therapeutic Advances in Neurological Disorders 2014, 7:52–59. 24409202 10.1177/1756285613495723 PMC3886380

[83] Flores-Torres MH, Christine CW, Bjornevik K, Molsberry SA, Hung AY, Healy BC, Blacker D, Schwarzschild MA, Ascherio A: Long-Term Intake of Folate, Vitamin B6, and Vitamin B12 and the Incidence of Parkinson’s Disease in a Sample of U.S. Women and Men. Movement Disorders 2023, 38:866–879. 36938854 10.1002/mds.29383 PMC12380053

[84] Schaffner A, Li X, Gomez-Llorente Y, Leandrou E, Memou A, Clemente N, Yao C, Afsari F, Zhi L, Pan N, : Vitamin B12 modulates Parkinson’s disease LRRK2 kinase activity through allosteric regulation and confers neuroprotection. Cell Research 2019, 29:313–329. 30858560 10.1038/s41422-019-0153-8 PMC6462009

[85] Dong L, Li Y-Z, An H-T, Wang Y-L, Chen S-H, Qian Y-J, Wang K, Zhen J-L, Fan Z, Gong X-L, : The E3 Ubiquitin Ligase c-Cbl Inhibits Microglia-Mediated CNS Inflammation by Regulating PI3K/Akt/NF-κB Pathway. CNS Neuroscience & Therapeutics 2016, 22:661–669. 27156691 10.1111/cns.12557 PMC6492864

[86] Wei Y, Lu M, Mei M, Wang H, Han Z, Chen M, Yao H, Song N, Ding X, Ding J, : Pyridoxine induces glutathione synthesis via PKM2-mediated Nrf2 transactivation and confers neuroprotection. Nature Communications 2020, 11:941. 10.1038/s41467-020-14788-x PMC7029000 32071304

[87] Galesic A, Pratt MR: Investigating the Effects of O-GlcNAc Modifications in Parkinson’s Disease Using Semisynthetic α-Synuclein. In Expressed Protein Ligation: Methods and Protocols. Edited by Vila-Perelló M. New York, NY: Springer US; 2020: 313–326 10.1007/978-1-0716-0434-2_15 PMC7143810 32144674

[88] Bilal B, Kirazlar M, Erdogan MA, Yigitturk G, Erbas O: Lacosamide exhibits neuroprotective effects in a rat model of Parkinson’s disease. Journal of Chemical Neuroanatomy 2023, 132:102311. 37442244 10.1016/j.jchemneu.2023.102311

[89] Kim JJ, Vitale D, Otani DV, Lian MM, Heilbron K, Aslibekyan S, Auton A, Babalola E, Bell RK, Bielenberg J, : Multi-ancestry genome-wide association meta-analysis of Parkinson’s disease. Nature Genetics 2023. 10.1038/s41588-023-01584-8 PMC10786718 38155330

[90] Lander E, Kruglyak L: Genetic dissection of complex traits: guidelines for interpreting and reporting linkage results. Nature Genetics 1995, 11:241–247. 7581446 10.1038/ng1195-241

[91] Sullivan KA, Lane M, Cashman M, Miller JI, Pavicic M, Walker AM, Cliff A, Romero J, Qin X, Lindquist J, : Digging deeper into GWAS signal using GRIN implicates additional genes contributing to suicidal behavior. medRxiv 2022:2022.2004.2020.22273895. 10.1038/s42003-024-06943-7 PMC11494055 39433874

[92] Guo Y, Huang Y, Hou L, Ma J, Chen C, Ai H, Huang L, Ren J: Genome-wide detection of genetic markers associated with growth and fatness in four pig populations using four approaches. Genetics Selection Evolution 2017, 49:21. 10.1186/s12711-017-0295-4 PMC5307927 28196480

[93] Corrà MF, Vila-Chã N, Sardoeira A, Hansen C, Sousa AP, Reis I, Sambayeta F, Damásio J, Calejo M, Schicketmueller A, : Peripheral neuropathy in Parkinson’s disease: prevalence and functional impact on gait and balance. Brain 2022, 146:225–236. 10.1093/brain/awac026 PMC9825570 35088837

[94] Tansey MG, Wallings RL, Houser MC, Herrick MK, Keating CE, Joers V: Inflammation and immune dysfunction in Parkinson disease. Nature Reviews Immunology 2022, 22:657–673. 10.1038/s41577-022-00684-6 PMC8895080 35246670

[95] Fujita M, Gao Z, Zeng L, McCabe C, White CC, Ng B, Green GS, Rozenblatt-Rosen O, Phillips D, Amir-Zilberstein L, : Cell subtype-specific effects of genetic variation in the Alzheimer’s disease brain. Nature Genetics 2024, 56:605–614. 38514782 10.1038/s41588-024-01685-y PMC12288883

[96] Bryois J, Calini D, Macnair W, Foo L, Urich E, Ortmann W, Iglesias VA, Selvaraj S, Nutma E, Marzin M, : Cell-type-specific cis-eQTLs in eight human brain cell types identify novel risk genes for psychiatric and neurological disorders. Nature Neuroscience 2022, 25:1104–1112. 35915177 10.1038/s41593-022-01128-z

[97] Nalls MA, Blauwendraat C, Vallerga CL, Heilbron K, Bandres-Ciga S, Chang D, Tan M, Kia DA, Noyce AJ, Xue A, : Identification of novel risk loci, causal insights, and heritable risk for Parkinson’s disease: a meta-analysis of genome-wide association studies. The Lancet Neurology 2019, 18:1091–1102. 31701892 10.1016/S1474-4422(19)30320-5 PMC8422160

